# Poly[(μ_5_-5-carboxylatotetrahydrofuran-2,3,4-tricarboxylic acid)sodium]

**DOI:** 10.1107/S160053680904269X

**Published:** 2009-10-23

**Authors:** Jie Xu, Wenxiang Chai, Jian Lin, Hongsheng Shi, Kangying Shu

**Affiliations:** a College of Materials Science and Engineering, China Jiliang University, Hangzhou 310018, People’s Republic of China

## Abstract

The search for the novel metal-organic frameworks (MOFs) materials using tetra­hydro­furan-2,3,4,5-tetra­carboxylic acid (THFTCA) as a versatile multi-carboxyl ligand, lead to the synthesis and the structure determination of the title compound, [Na(H_3_THFTCA)] or [Na(C_8_H_7_O_9_)]_*n*_, which was obtained by a solution reaction at room temperature. The ligand is mono-deprotonated, coordinating five sodium ions through one furan oxygen atom and six carboxyl oxygen atoms. The sodium ion exhibits a distorted penta­gonal-bipyramidal NaO_7_ geometry consisting of seven O atoms derived from five surrounding ligands. Two adjacent pentagonal bipyramids share an O—O edge, forming a dinuclear sodium cluster. Finally, these clusters are effectively linked by the carboxyl groups of THFTCA ligands, forming a firm metal organic framework and O—H⋯O hydrogen bonds contribute to the crystal packing.

## Related literature

For potential applications of metal-organic frameworks (MOFs), see: Moulton & Zaworotko (2001 ▶); Bradshaw *et al.* (2007 ▶). Self-assembly of selected ligands around *d*-transition metal ions is a widespread method for obtaining novel MOF structures, see: Leininger *et al.* (2000 ▶). In contrast, the *s*-elements are more flexible of their coordination behaviour, and maybe present in more various structures, see: Lu *et al.* (2007 ▶). For related MOF materials constructed from the THFTCA ligand, see: Hanson *et al.* (2004 ▶); Thuéry *et al.* (2004 ▶); Ai *et al.* (2008 ▶); Wang & Sevov (2007 ▶); Wang *et al.* (2007 ▶); Lü (2008 ▶). For related *s*-elements and THFTCA ligand compound structures, see: Barnes & Paton (1984 ▶) for Cs^+^ and Ca^2+;^ Barnes (2002 ▶) for Na^+^; Paul & Martin (1967 ▶) for Rb^+^.
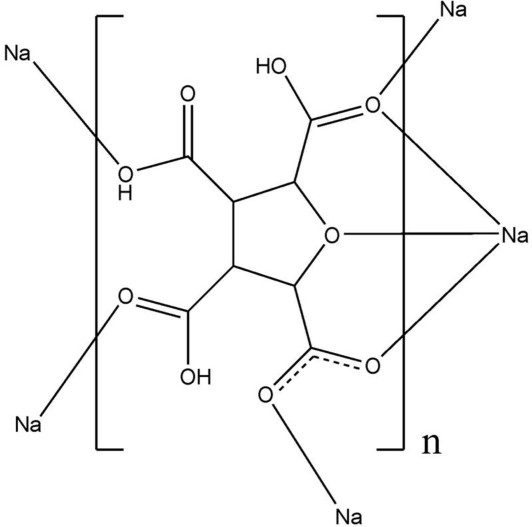


## Experimental

### 

#### Crystal data

[Na(C_8_H_7_O_9_)]
*M* *_r_* = 270.13
Monoclinic, 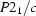

*a* = 8.0663 (16) Å
*b* = 13.417 (3) Å
*c* = 9.7358 (19) Å
β = 109.90 (3)°
*V* = 990.7 (3) Å^3^
*Z* = 4
Mo *K*α radiation
μ = 0.20 mm^−1^
*T* = 296 K
0.41 × 0.28 × 0.10 mm

#### Data collection

Rigaku R-AXIS RAPID diffractometer
Absorption correction: multi-scan (*ABSCOR*; Higashi, 1995 ▶) *T*_min_ = 0.921, *T*_max_ = 0.980
9567 measured reflections
2263 independent reflections
2095 reflections with *I* > 2σ(*I*)
*R*_int_ = 0.018

#### Refinement

*R*[*F* ^2^ > 2σ(*F* ^2^)] = 0.032
*wR*(*F* ^2^) = 0.090
*S* = 1.09
2263 reflections
164 parameters
H-atom parameters constrained
Δρ_max_ = 0.39 e Å^−3^
Δρ_min_ = −0.32 e Å^−3^

### 

Data collection: *PROCESS-AUTO* (Rigaku, 1998 ▶); cell refinement: *PROCESS-AUTO*; data reduction: *CrystalStructure* (Rigaku/MSC, 2004 ▶); program(s) used to solve structure: *SHELXS97* (Sheldrick, 2008 ▶); program(s) used to refine structure: *SHELXL97* (Sheldrick, 2008 ▶); molecular graphics: *SHELXTL* (Sheldrick, 2008 ▶); software used to prepare material for publication: *SHELXTL*.

## Supplementary Material

Crystal structure: contains datablocks I, global. DOI: 10.1107/S160053680904269X/kp2234sup1.cif

Structure factors: contains datablocks I. DOI: 10.1107/S160053680904269X/kp2234Isup2.hkl

Additional supplementary materials:  crystallographic information; 3D view; checkCIF report

## Figures and Tables

**Table 1:** Hydrogen-bond geometry (Å, °)

*D*—H⋯*A*	*D*—H	H⋯*A*	*D*⋯*A*	*D*—H⋯*A*
O3—H3⋯O9^i^	0.84	1.70	2.5395 (15)	173
O5—H5⋯O6^ii^	0.84	1.83	2.6468 (16)	165
O7—H7⋯O8^iii^	0.84	1.68	2.5169 (14)	171

Symmetry codes: (i) 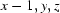; (ii) 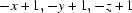; (iii) 
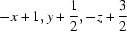
.

**Fig. 1.**
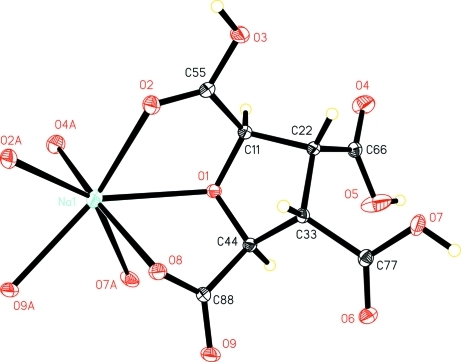
Structure and labeling of the title compound, with displacement ellipsoids drawn at the 30% probability level and H atoms shown as small spheres of arbitrary radii.

**Fig. 2.**
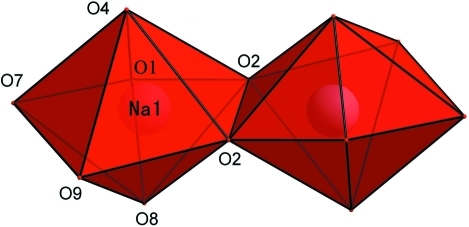
The distorted pentagonal bipyramid NaO7 coordination polyhedron with the common edge O2-O2 shows a dinuclear sodium unit.

**Fig. 3.**
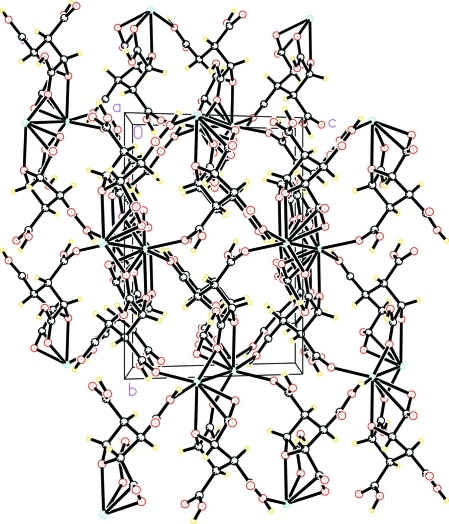
The packing diagram viewed along the axis a, Na: blue diagonal; O: red inner dot; C: black circles; and H: small green circles.

## supplementary crystallographic information

### Comment

Metal organic frameworks (MOFs) have attracted a great deal of interest owing to the ability to tune their porosity and the functionalities that are incorporated within the framework scaffolds. As a result, numerous MOFs have been engineered for a number of potential applications, including gas storage, nonlinear optics, catalysis, and so on. (Moulton *et al.* 2001; Bradshaw *et al.* 2007) Usually, highly directional coordination bonds are adopted in the design of MOFs, and self-assembly of selected ligands around d-transition metal ions is now a widespread method for obtaining novel MOFs structure (Leininger *et al.* 2000). In contrast, the s-elements are more flexible of their coordination behaviour, and maybe present in more various structures (Lu *et al.* 2007). On the other hand, for the complex ligand with large numbers of potential binding sites, such as, tetrahydrofuran-2, 3, 4, 5-tetracarboxylic acid, it is difficult to predict the final structure. Therefore, the investigation of these complex ligands might provide novel MOFs with interesting structural topology. However, reports on THFTCA are rare (Hanson *et al.* 2004). Here, we report a three-dimensional MOFs compound Na(H_3_THFTCA) (I), which is assembled from THFTCA and sodium ion.

The title compound has a three-dimensional framework structure constructed by mono deprotonated THFTCA ligand; the asymmetric unit contains one full chiral THFTCA ligand and one sodium atom (Fig. 1). The THFTCA ligand coordinates the sodium ion with its furan oxygen atom and two adjacent carboxyl oxygen atoms, while its four carboxyl groups also grasp the neighbouring four sodium ions (Scheme 1). Thus, the sodium ion is located in a distorted pentagonal bipyramid NaO_7_, coordinated by seven O atoms from  the five ligands. The two pentagonal bipyramids are fused via a common eadge O2—O2, generating a dinuclear sodium cluster with an an inversion centre at the midpoint of eadge O2—O2 (Fig. 2). The title compound crystallizes in the centrosymmetric space group P21/c implying the presence of a racemate (1:1) in the crystal. The dinuclear sodium clusters are connected by carboxyl groups of THFTCA ligand. The crystal packing includes firm framework of multi-carboxyl ligand and sodium ion connected by hydrogen bonds of O—H···O (Table 1, Fig. 3).

### Experimental

All chemicals were obtained from commercial sources and were used as obtained. The title compound was handily synthesized by a solution reaction from H4THFTCA. H_4_THFTCA (154 mg, 0.6 mmol) and NaOH (25 mg, 0.6 mmol) was dissolved in 10 ml of water. To this solution was added a 5 ml aqueous solution of Nd(NO_3_)_3_. 6H_2_O (89 mg, 0.2 mmol) at room temperature. Amount of colourless crystals were obtained after the filtration was slowly evaporated at room temperature for several days.

### Refinement

The structure was solved using direct methods and refined by full-matrix least-squares techniques. All non-hydrogen atoms were assigned anisotropic displacement parameters in the refinement. All hydrogen atoms were added at calculated positions and refined using a riding model.(Sheldrick *et al.*, 2008).

### Crystal data

**Table:**

[Na(C_8_H_7_O_9_)]	*F*(000) = 552
*M**_r_* = 270.13	*D*_x_ = 1.811 Mg m^−^^3^
Monoclinic, *P*2_1_/*c*	Mo *K*α radiation, λ = 0.71075 Å
Hall symbol: -P 2ybc	Cell parameters from 8534 reflections
*a* = 8.0663 (16) Å	θ = 3.0–27.4°
*b* = 13.417 (3) Å	µ = 0.20 mm^−^^1^
*c* = 9.7358 (19) Å	*T* = 296 K
β = 109.90 (3)°	Platelet, colorless
*V* = 990.7 (3) Å^3^	0.41 × 0.28 × 0.10 mm
*Z* = 4	

### Data collection

**Table:**

Rigaku R-AXIS RAPID diffractometer	2263 independent reflections
Radiation source: fine-focus sealed tube	2095 reflections with *I* > 2σ(*I*)
graphite	*R*_int_ = 0.018
Detector resolution: 14.6306 pixels mm^-1^	θ_max_ = 27.4°, θ_min_ = 3.0°
CCD_Profile_fitting scans	*h* = −10→10
Absorption correction: multi-scan (*ABSCOR*; Higashi, 1995)	*k* = −17→17
*T*_min_ = 0.921, *T*_max_ = 0.980	*l* = −12→12
9567 measured reflections	

### Refinement

**Table:**

Refinement on *F*^2^	Secondary atom site location: difference Fourier map
Least-squares matrix: full	Hydrogen site location: inferred from neighbouring sites
*R*[*F*^2^ > 2σ(*F*^2^)] = 0.032	H-atom parameters constrained
*wR*(*F*^2^) = 0.090	*w* = 1/[σ^2^(*F*_o_^2^) + (0.0479*P*)^2^ + 0.4297*P*] where *P* = (*F*_o_^2^ + 2*F*_c_^2^)/3
*S* = 1.09	(Δ/σ)_max_ < 0.001
2263 reflections	Δρ_max_ = 0.39 e Å^−^^3^
164 parameters	Δρ_min_ = −0.31 e Å^−^^3^
0 restraints	Extinction correction: *SHELXL*, Fc^*^=kFc[1+0.001xFc^2^λ^3^/sin(2θ)]^-1/4^
Primary atom site location: structure-invariant direct methods	Extinction coefficient: 0.034 (3)

### Special details

**Table:**

Geometry. All e.s.d.'s (except the e.s.d. in the dihedral angle between two l.s. planes) are estimated using the full covariance matrix. The cell e.s.d.'s are taken into account individually in the estimation of e.s.d.'s in distances, angles and torsion angles; correlations between e.s.d.'s in cell parameters are only used when they are defined by crystal symmetry. An approximate (isotropic) treatment of cell e.s.d.'s is used for estimating e.s.d.'s involving l.s. planes.
Refinement. Refinement of *F*^2^ against ALL reflections. The weighted *R*-factor *wR* and goodness of fit *S* are based on *F*^2^, conventional *R*-factors *R* are based on *F*, with *F* set to zero for negative *F*^2^. The threshold expression of *F*^2^ > σ(*F*^2^) is used only for calculating *R*-factors(gt) *etc*. and is not relevant to the choice of reflections for refinement. *R*-factors based on *F*^2^ are statistically about twice as large as those based on *F*, and *R*- factors based on ALL data will be even larger.

### Fractional atomic coordinates and isotropic or equivalent isotropic displacement parameters (Å^2^)

**Table:**

	*x*	*y*	*z*	*U*_iso_*/*U*_eq_	
Na1	0.16477 (7)	0.01271 (4)	0.39826 (6)	0.02678 (17)	
O1	0.19961 (11)	0.20181 (7)	0.39121 (9)	0.0179 (2)	
O2	−0.03876 (14)	0.10910 (7)	0.48915 (13)	0.0313 (3)	
O3	−0.17693 (14)	0.25311 (8)	0.49566 (13)	0.0333 (3)	
H3	−0.2454	0.2189	0.5252	0.033*	
O4	0.02774 (14)	0.47842 (9)	0.33101 (13)	0.0362 (3)	
O5	0.31727 (15)	0.46326 (10)	0.42755 (16)	0.0506 (4)	
H5	0.3204	0.5163	0.3827	0.051*	
O6	0.60941 (12)	0.37079 (7)	0.68721 (11)	0.0251 (2)	
O7	0.39834 (13)	0.43913 (7)	0.75861 (11)	0.0281 (2)	
H7	0.4778	0.4798	0.8028	0.028*	
O8	0.39053 (12)	0.07324 (7)	0.60685 (11)	0.0234 (2)	
O9	0.63546 (12)	0.13823 (7)	0.58976 (12)	0.0272 (2)	
C11	0.05765 (15)	0.26035 (9)	0.40594 (13)	0.0167 (2)	
H11	−0.0167	0.2819	0.3091	0.017*	
C22	0.14282 (15)	0.34871 (9)	0.50496 (13)	0.0159 (2)	
H22	0.0684	0.3706	0.5593	0.016*	
C33	0.31740 (15)	0.30122 (8)	0.60199 (12)	0.0147 (2)	
H33	0.2877	0.2598	0.6734	0.015*	
C44	0.36719 (15)	0.23349 (8)	0.49541 (12)	0.0149 (2)	
H44	0.4337	0.2705	0.4470	0.015*	
C55	−0.05776 (16)	0.19811 (10)	0.46899 (14)	0.0202 (3)	
C66	0.15670 (16)	0.43746 (9)	0.41175 (14)	0.0197 (3)	
C77	0.45783 (16)	0.37456 (9)	0.68641 (13)	0.0178 (2)	
C88	0.47422 (15)	0.14121 (9)	0.56952 (13)	0.0163 (2)	

### Atomic displacement parameters (Å^2^)

**Table:**

	*U*^11^	*U*^22^	*U*^33^	*U*^12^	*U*^13^	*U*^23^
Na1	0.0233 (3)	0.0215 (3)	0.0317 (3)	−0.0036 (2)	0.0044 (2)	−0.0026 (2)
O1	0.0145 (4)	0.0192 (4)	0.0191 (4)	0.0008 (3)	0.0043 (3)	−0.0038 (3)
O2	0.0268 (5)	0.0179 (5)	0.0538 (7)	0.0001 (4)	0.0195 (5)	0.0058 (4)
O3	0.0272 (5)	0.0212 (5)	0.0619 (7)	−0.0005 (4)	0.0286 (5)	0.0012 (5)
O4	0.0261 (5)	0.0321 (6)	0.0424 (6)	0.0028 (4)	0.0012 (5)	0.0170 (5)
O5	0.0222 (6)	0.0509 (7)	0.0725 (9)	−0.0036 (5)	0.0078 (6)	0.0436 (7)
O6	0.0173 (5)	0.0233 (5)	0.0327 (5)	−0.0026 (4)	0.0059 (4)	0.0003 (4)
O7	0.0258 (5)	0.0243 (5)	0.0355 (5)	−0.0092 (4)	0.0123 (4)	−0.0145 (4)
O8	0.0186 (4)	0.0189 (4)	0.0319 (5)	0.0009 (4)	0.0077 (4)	0.0092 (4)
O9	0.0158 (4)	0.0232 (5)	0.0433 (6)	0.0031 (4)	0.0112 (4)	0.0073 (4)
C11	0.0146 (5)	0.0151 (5)	0.0195 (5)	0.0006 (4)	0.0047 (4)	0.0002 (4)
C22	0.0140 (5)	0.0138 (5)	0.0199 (5)	−0.0006 (4)	0.0056 (4)	0.0000 (4)
C33	0.0154 (5)	0.0128 (5)	0.0161 (5)	−0.0004 (4)	0.0058 (4)	0.0007 (4)
C44	0.0142 (5)	0.0142 (5)	0.0164 (5)	−0.0006 (4)	0.0052 (4)	0.0012 (4)
C55	0.0152 (5)	0.0188 (6)	0.0256 (6)	−0.0023 (5)	0.0057 (5)	−0.0010 (5)
C66	0.0190 (6)	0.0151 (5)	0.0238 (6)	0.0001 (4)	0.0057 (5)	0.0014 (4)
C77	0.0197 (6)	0.0149 (5)	0.0171 (5)	−0.0017 (4)	0.0042 (4)	0.0021 (4)
C88	0.0158 (5)	0.0149 (5)	0.0182 (5)	0.0009 (4)	0.0058 (4)	−0.0008 (4)

### Geometric parameters (Å, °)

**Table:**

Na1—O4^i^	2.2903 (14)	O7—C77	1.3056 (16)
Na1—O8	2.3626 (14)	O7—Na1^vi^	2.7495 (13)
Na1—O2^ii^	2.3800 (12)	O7—H7	0.8400
Na1—O2	2.4778 (13)	O8—C88	1.2595 (15)
Na1—O1	2.5561 (12)	O9—C88	1.2480 (15)
Na1—O9^iii^	2.5658 (12)	O9—Na1^iii^	2.5658 (12)
Na1—O7^iv^	2.7495 (13)	C11—C55	1.5264 (17)
O1—C11	1.4354 (14)	C11—C22	1.5349 (16)
O1—C44	1.4502 (14)	C11—H11	0.9734
O2—C55	1.2114 (16)	C22—C66	1.5242 (17)
O2—Na1^ii^	2.3800 (12)	C22—C33	1.5413 (16)
O3—C55	1.3061 (16)	C22—H22	0.9710
O3—H3	0.8400	C33—C77	1.5143 (16)
O4—C66	1.2021 (17)	C33—C44	1.5322 (16)
O4—Na1^v^	2.2903 (14)	C33—H33	0.9811
O5—C66	1.2982 (17)	C44—C88	1.5410 (16)
O5—H5	0.8402	C44—H44	0.9638
O6—C77	1.2210 (16)		
			
O4^i^—Na1—O8	167.22 (5)	O1—C11—C22	106.48 (9)
O4^i^—Na1—O2^ii^	93.25 (5)	C55—C11—C22	111.95 (10)
O8—Na1—O2^ii^	99.51 (4)	O1—C11—H11	108.4
O4^i^—Na1—O2	98.11 (5)	C55—C11—H11	107.0
O8—Na1—O2	85.65 (4)	C22—C11—H11	112.0
O2^ii^—Na1—O2	75.83 (4)	C66—C22—C11	109.71 (10)
O4^i^—Na1—O1	102.56 (4)	C66—C22—C33	116.80 (10)
O8—Na1—O1	67.81 (3)	C11—C22—C33	100.71 (9)
O2^ii^—Na1—O1	139.65 (4)	C66—C22—H22	105.8
O2—Na1—O1	65.41 (3)	C11—C22—H22	110.4
O4^i^—Na1—O9^iii^	95.32 (5)	C33—C22—H22	113.4
O8—Na1—O9^iii^	86.78 (4)	C77—C33—C44	115.65 (10)
O2^ii^—Na1—O9^iii^	78.30 (4)	C77—C33—C22	114.99 (10)
O2—Na1—O9^iii^	151.39 (4)	C44—C33—C22	103.04 (9)
O1—Na1—O9^iii^	135.36 (4)	C77—C33—H33	107.5
O4^i^—Na1—O7^iv^	85.22 (5)	C44—C33—H33	109.1
O8—Na1—O7^iv^	83.50 (4)	C22—C33—H33	106.0
O2^ii^—Na1—O7^iv^	149.44 (4)	O1—C44—C33	104.52 (9)
O2—Na1—O7^iv^	134.65 (4)	O1—C44—C88	109.41 (9)
O1—Na1—O7^iv^	69.72 (3)	C33—C44—C88	113.18 (9)
O9^iii^—Na1—O7^iv^	71.49 (4)	O1—C44—H44	110.5
C11—O1—C44	110.80 (9)	C33—C44—H44	110.1
C11—O1—Na1	116.21 (7)	C88—C44—H44	109.1
C44—O1—Na1	110.91 (6)	O2—C55—O3	125.86 (12)
C55—O2—Na1^ii^	134.52 (9)	O2—C55—C11	122.93 (12)
C55—O2—Na1	121.28 (9)	O3—C55—C11	111.21 (11)
Na1^ii^—O2—Na1	104.17 (4)	O4—C66—O5	124.19 (13)
C55—O3—H3	111.9	O4—C66—C22	121.59 (12)
C66—O4—Na1^v^	151.37 (11)	O5—C66—C22	114.22 (11)
C66—O5—H5	111.7	O6—C77—O7	125.09 (12)
C77—O7—Na1^vi^	149.49 (8)	O6—C77—C33	122.61 (11)
C77—O7—H7	110.5	O7—C77—C33	112.29 (11)
Na1^vi^—O7—H7	97.2	O9—C88—O8	124.29 (11)
C88—O8—Na1	109.44 (8)	O9—C88—C44	119.16 (11)
C88—O9—Na1^iii^	129.58 (8)	O8—C88—C44	116.55 (10)
O1—C11—C55	110.94 (10)		
			
O4^i^—Na1—O1—C11	77.60 (8)	C11—C22—C33—C77	164.09 (10)
O8—Na1—O1—C11	−111.21 (8)	C66—C22—C33—C44	−81.34 (12)
O2^ii^—Na1—O1—C11	−33.01 (10)	C11—C22—C33—C44	37.35 (11)
O2—Na1—O1—C11	−15.66 (7)	C11—O1—C44—C33	12.61 (12)
O9^iii^—Na1—O1—C11	−171.25 (7)	Na1—O1—C44—C33	−117.99 (7)
O7^iv^—Na1—O1—C11	157.62 (8)	C11—O1—C44—C88	134.10 (10)
O4^i^—Na1—O1—C44	−154.68 (7)	Na1—O1—C44—C88	3.50 (10)
O8—Na1—O1—C44	16.50 (7)	C77—C33—C44—O1	−157.88 (9)
O2^ii^—Na1—O1—C44	94.70 (9)	C22—C33—C44—O1	−31.56 (11)
O2—Na1—O1—C44	112.05 (8)	C77—C33—C44—C88	83.16 (12)
O9^iii^—Na1—O1—C44	−43.54 (9)	C22—C33—C44—C88	−150.52 (10)
O7^iv^—Na1—O1—C44	−74.67 (7)	Na1^ii^—O2—C55—O3	−7.4 (2)
O4^i^—Na1—O2—C55	−87.32 (12)	Na1—O2—C55—O3	170.75 (11)
O8—Na1—O2—C55	80.39 (11)	Na1^ii^—O2—C55—C11	173.27 (9)
O2^ii^—Na1—O2—C55	−178.65 (14)	Na1—O2—C55—C11	−8.56 (18)
O1—Na1—O2—C55	12.84 (10)	O1—C11—C55—O2	−6.66 (17)
O9^iii^—Na1—O2—C55	155.50 (10)	C22—C11—C55—O2	−125.45 (14)
O7^iv^—Na1—O2—C55	3.96 (14)	O1—C11—C55—O3	173.94 (10)
O4^i^—Na1—O2—Na1^ii^	91.33 (5)	C22—C11—C55—O3	55.15 (14)
O8—Na1—O2—Na1^ii^	−100.96 (5)	Na1^v^—O4—C66—O5	73.4 (3)
O2^ii^—Na1—O2—Na1^ii^	0.0	Na1^v^—O4—C66—C22	−106.3 (2)
O1—Na1—O2—Na1^ii^	−168.51 (6)	C11—C22—C66—O4	65.15 (16)
O9^iii^—Na1—O2—Na1^ii^	−25.85 (10)	C33—C22—C66—O4	178.86 (12)
O7^iv^—Na1—O2—Na1^ii^	−177.39 (5)	C11—C22—C66—O5	−114.57 (14)
O4^i^—Na1—O8—C88	3.2 (2)	C33—C22—C66—O5	−0.86 (17)
O2^ii^—Na1—O8—C88	−179.35 (8)	Na1^vi^—O7—C77—O6	−151.33 (12)
O2—Na1—O8—C88	−104.52 (9)	Na1^vi^—O7—C77—C33	27.3 (2)
O1—Na1—O8—C88	−39.34 (8)	C44—C33—C77—O6	−10.65 (16)
O9^iii^—Na1—O8—C88	103.09 (9)	C22—C33—C77—O6	−130.65 (12)
O7^iv^—Na1—O8—C88	31.37 (8)	C44—C33—C77—O7	170.70 (10)
C44—O1—C11—C55	−110.30 (11)	C22—C33—C77—O7	50.70 (14)
Na1—O1—C11—C55	17.46 (12)	Na1^iii^—O9—C88—O8	29.64 (19)
C44—O1—C11—C22	11.74 (12)	Na1^iii^—O9—C88—C44	−150.88 (8)
Na1—O1—C11—C22	139.51 (7)	Na1—O8—C88—O9	−120.38 (12)
O1—C11—C22—C66	93.14 (11)	Na1—O8—C88—C44	60.13 (12)
C55—C11—C22—C66	−145.46 (10)	O1—C44—C88—O9	138.01 (11)
O1—C11—C22—C33	−30.58 (11)	C33—C44—C88—O9	−105.88 (13)
C55—C11—C22—C33	90.82 (11)	O1—C44—C88—O8	−42.47 (14)
C66—C22—C33—C77	45.40 (14)	C33—C44—C88—O8	73.63 (13)

Symmetry codes: (i) −*x*, *y*−1/2, −*z*+1/2; (ii) −*x*, −*y*, −*z*+1; (iii) −*x*+1, −*y*, −*z*+1; (iv) *x*, −*y*+1/2, *z*−1/2; (v) −*x*, *y*+1/2, −*z*+1/2; (vi) *x*, −*y*+1/2, *z*+1/2.

### Hydrogen-bond geometry (Å, °)

**Table:**

*D*—H···*A*	*D*—H	H···*A*	*D*···*A*	*D*—H···*A*
O3—H3···O9^vii^	0.84	1.70	2.5395 (15)	173
O5—H5···O6^viii^	0.84	1.83	2.6468 (16)	165
O7—H7···O8^ix^	0.84	1.68	2.5169 (14)	171

Symmetry codes: (vii) *x*−1, *y*, *z*; (viii) −*x*+1, −*y*+1, −*z*+1; (ix) −*x*+1, *y*+1/2, −*z*+3/2.
